# Supplementary material for: Effects of outdoor play on body composition and physical performance in children: the Yamanashi Adjunct study of the Japan Environment and Children’s Study
Source: PLoS One. 2025 Feb 10;20(2):e0317925. doi: 10.1371/journal.pone.0317925 (PMC11809897; doi:10.1371/journal.pone.0317925)
Supplement: Attachment — Submitted filename: Response_to_Reviewers.docx [file pone.0317925.s002.docx]

**Responses to the Reviewers’ Comments**

**Reviewer 1**

**Comment１:**

Why were the data of 8-year-old participants isolated and assessed? The authors should describe the reason in the Discussion section.

**Response:**

Thank you for your question. The Japan Environment and Children's Study is a nationwide birth cohort study on children's health and development that follows children from before birth to approximately 20 years of age and is conducted under the initiative of the Ministry of the Environment. Specifically, document surveys are conducted every 6 months after birth, and face-to-face surveys are conducted every few years. It was decided from the planning stage that a face-to-face survey with uniform content would be conducted nationwide at 8 years of age. In addition, our Koshin Unit Center independently conducted surveys on postural stability, dental and oral surgery surveys, and intestinal bacteria tests. This study used these data and targeted 8-year-old children. Some of these points have been added in the Materials and Methods section (lines 116–119).

Revised text

“Specifically, document surveys are conducted every 6 months after birth, and face-to-face surveys are conducted every few years. It was decided from the planning stage that a face-to-face survey with uniform content would be conducted nationwide at 8 years of age. In addition, we independently conducted adjunct surveys.”

**Comment 2:**

Why were 846 participants excluded from the 1,340 participants? The excluded number is too many to evaluate the overall trends of this study statistically. The exclusion criteria should be described in detail.

**Response:**

Thank you for pointing this out. We agree with you. The main reasons for the low collection rate of sports test results were that the timing of the survey coincided with the coronavirus disease epidemic and that many elementary schools did not have sports tests. The timing of the survey could not be changed because it was to be conducted at a predetermined period of the Ministry of the Environment’s cohort program. This information has been added to the paragraph on study limitations (lines 365–369).

Revised text

“Other reasons for the low collection rate of sports test results were that the timing of the survey coincided with the coronavirus disease epidemic and that many elementary schools did not have sports tests. The timing of the survey could not be changed because it was to be conducted at a predetermined period of the Ministry of the Environment’s cohort program.”

**Comment 3:**

The definition of outdoor playtime should be described in detail. Does the outdoor playtime mean an average of a week or every day?

**Response:**

Thank you for your question. We used the results of a survey in which parents were asked if their children played outside for at least 1 hour per day (lines 152–153). Although this was a survey and may not be accurate in some respects, we believe that it is an acceptable form of questioning for making a determination of whether children play outside more or prefer to play indoors.

Revised text

“The amount of time spent playing outside was investigated by asking the participants’ parents via the survey whether their children play outside for an average of at least 1 hour per day.”

**Comment 4:**

The reviewer recommends that the statistical analysis of correlations between sports tests and body composition should also be conducted by the total participants, not by boys and girls differentially.

**Response:**

Thank you for your question. All the study results include not only boys and girls separately but also the total participants (Table 3a, lines 274–276).

Revised text

**Table 3. Correlations between sports test results and body composition.**

a. Total

|  | | **Sports test** | | | |
| --- | --- | --- | --- | --- | --- |
|  | | **HG** | **50S** | **SLJ** | **20SR** |
| **Phase angle** | **RU** | 0.1925** | 0.0665 | 0.0944* | 0.0741 |
|  | **LU** | 0.1463** | 0.0515 | 0.0325 | 0.0878 |
|  | **RL** | 0.2784** | -0.2680** | 0.2313** | 0.2318** |
|  | **LL** | 0.2741** | -0.2546** | 0.2234** | 0.2335** |
| **MW** | **trunk** | 0.3915** | 0.0178 | 0.0193 | -0.0080 |
|  | **RU** | 0.3873** | 0.0276 | 0.0329 | -0.0905* |
|  | **LU** | 0.3961** | 0.0147 | 0.0413 | -0.0435 |
|  | **RL** | 0.4480** | -0.0295 | 0.0879 | -0.0221 |
|  | **LL** | 0.4532** | -0.0174 | 0.0808 | -0.0306 |

**Reviewer 2**

**Comment 1:**

Correct the word "splinting" in page 8 Line6 to "sprinting"

**Response:**

Thank you for pointing this out. The spelling of “sprinting” has been corrected (line 145).

Revised text

“In Japan, sports tests for primary school children are annually conducted nationwide according to uniform standards, and data on the 50 m sprinting time (50S), standing long jump distance (SLJ), 20 m shuttle run test (20SR), and hand grip strength (HG) were used from these tests.”

**Comment 2:**

Test of normality should be clarified in "Statistical analysis"

**Response:**

A description of normality has been added to the Materials and Methods section (lines 155–156). Since most data were normally distributed, t-tests and Pearson correlation coefficients were used in the statistical analyses.

Revised text

“Most data were normally distributed; thus, t-tests and Pearson correlation coefficients were used in the statistical analyses.”

**Comment 3:**

In "Discussion", some study limitations should be mentioned, such as determining the type of outdoor activities, whether these children live in urban or rural areas, did the child perform these outdoor activities daily, whether during school period or vacation and what is the maximal time for these outdoor activities.

**Response:**

Thank you for your comments. This study was based on data from a survey of parents who were asked “Do you play outside for at least 1 hour per day? The participants were recruited from all over our prefecture, including those living in mountainous areas and residential areas. As you pointed out, it is necessary to examine the content of outdoor play and the area (rural or urban) in which they live. However, in Yamanashi Prefecture, there are no extremely rural areas or extremely urban areas; thus, we do not believe that there are significant differences in outdoor play and lifestyles in the areas where the children live.
